# Prosaposin PS18 reduces dopaminergic neurodegeneration in a 6-hydroxydopamine rat model of Parkinson’s disease

**DOI:** 10.1038/s41598-023-35274-6

**Published:** 2023-05-19

**Authors:** Kuo-Jen Wu, Tsai-Wei Hung, Yu-Syuan Wang, Yun-Hsiang Chen, Eun-Kyung Bae, Seong-Jin Yu

**Affiliations:** 1 Center for Neuropsychiatric Research, National Health Research Institutes, 35 Keyan Road, Zhunan, 35053 Miaoli Taiwan; 2 Department of Life Science, Fu-Jen Catholic University, New Taipei City, Taiwan

**Keywords:** Biochemistry, Drug discovery, Neuroscience, Neurology, Pathogenesis

## Abstract

Saposin and its precursor prosaposin are endogenous proteins with neurotrophic and anti-apoptotic properties. Prosaposin or its analog prosaposin-derived 18-mer peptide (PS18) reduced neuronal damage in hippocampus and apoptosis in stroke brain. Its role in Parkinson’s disease (PD) has not been well characterized. This study aimed to examine the physiological role of PS18 in 6-hydroxydopamine (6-OHDA) cellular and animal models of PD. We found that PS18 significantly antagonized 6-OHDA -mediated dopaminergic neuronal loss and TUNEL in rat primary dopaminergic neuronal culture. In SH-SY5Y cells overexpressing the secreted ER calcium—monitoring proteins, we found that PS18 significantly reduced thapsigargin and 6-OHDA-mediated ER stress. The expression of prosaposin and the protective effect of PS18 were next examined in hemiparkinsonian rats. 6-OHDA was unilaterally administered to striatum. The expression of prosaposin was transiently upregulated in striatum on D3 (day 3) after lesioning and returned below the basal level on D29. The 6-OHDA-lesioned rats developed bradykinesia and an increase in methamphetamine-mediated rotation, which was antagonized by PS18. Brain tissues were collected for Western blot, immunohistochemistry, and qRTPCR analysis. Tyrosine hydroxylase immunoreactivity was significantly reduced while the expressions of PERK, ATF6, CHOP, and BiP were upregulated in the lesioned nigra; these responses were significantly antagonized by PS18. Taken together, our data support that PS18 is neuroprotective in cellular and animal models of PD. The mechanisms of protection may involve anti-ER stress.

## Introduction

Parkinson’s disease (PD) is the second most common neurodegenerative disease worldwide. It is characterized by a progressive loss of dopaminergic neurons in the midbrain, which leads to major clinical symptoms of bradykinesia, postural instability, rigidity, resting tremor, and mental illness^1,2^. The mechanisms of neuronal degeneration in PD involve endoplasmic reticulum stress (ER stress), oxidative stress, apoptosis, inflammation, genetic factors, and others. Levodopa (L-DOPA) is the most commonly used treatment for PD. L-DOPA increases dopamine production in brain and reduces the PD -related symptoms. However, L-DOPA cannot stop the progression of degeneration. In addition, patients often develop multiple side effects, including dyskinesia or psychosis, after long-term use of L-DOPA. Therefore, non-dopaminergic pharmacological treatments for PD are urgently needed.

Saposin is an acronym for sphingolipid activator protein. There are four saposins in the saposin family (i.e., saposin A–D). They are heat-stable glycoproteins derived from the precursor prosaposin, a 517 amino acid protein. Saposin and prosaposin are neurotrophic and anti-apoptotic^3^. Prosaposin is highly expressed in brain^4^ and mediates neurotrophic function through N-terminal^5^. The N-terminal domain of saposin C enhanced neurite outgrowth and prevented neuronal death^6^.

Prosaposin is highly expressed in nigra and striatum^7^. Mutation of the prosaposin gene has been linked to PD in patients^8^. Prosaposin mutations in the saposin D domain have been considered to associate with α-synuclein aggregation and dopaminergic neurodegeneration in the substantia nigra^8^.

The neuroprotective action of prosaposin has been reported, mainly in the non-dopaminergic neurons. Intracerebroventricular infusion of prosaposin reduced hippocampal CA1 neuronal damage in stroke gerbils^9^. A similar protective effect was found in prosaposin analog PS18, a prosaposin-derived 18-mer peptide (LSELIINNATEELLIKGL). PS18 prevented apoptosis in hippocampal neurons through the up-regulation of mitochondrial anti-apoptotic factor Bcl-x(L) in stroke brain^10^. The protective action of prosaposin or PS18 in nigrostriatal dopaminergic neurons is still not well characterized.

The endoplasmic reticulum (ER) is the primary intracellular reservoir for calcium (Ca^2+^). Reducing ER Ca^2+^ causes ER stress. Recent studies have shown that ER calcium homeostasis can be monitored through the secreted ER calcium–monitoring proteins (or SERCaMPs) in SY5Y cells^11,12^. SERCaMPs were designed based on the structure of an ER stress-sensitive C-terminal ASRTDL sequence, which is essential for the ER stress-mediated secretion^13^. A Gaussian luciferase (GLuc) reporter was added to ASRTDL to form GLuc-SERCaMP. The release of GLuc-SERCaMP is responsive to the ER stressor thapsigargin (Tg) or methamphetamine (Meth). ER stress can be quantified by measuring GLuc activity in cell culture^11,12^. GLuc-SERCaMP has also been used in vivo to monitor ER stress in brain^14^ during ischemic stroke and liver after fat diets^15^ and to screen therapeutics for treating ER stress^16^.

In this study, we examined the protective actions of PS18 in cellular and rat PD models. Here we reported that PS18 reduced 6-OHDA -mediated dopaminergic neuronal loss and apoptosis in rat primary ventromesencephalic (VM) culture. PS18 antagonized Tg and 6-OHDA -mediated GLuc-SERCaMP release in SY5Y cell culture. In addition, 6-OHDA time-dependently regulated the expression of prosaposin in brain. Intracerebroventricular administration of PS19 improved behavioral function in the 6-OHDA-lesioned rats, increased tyrosine hydroxylase immunoreactivity, and reduced the expression of ER stress markers in the lesioned brain. Our data support the notion that PS18 protects against PD-mediated neurodegeneration.

## Results

### PS18 induced neuroprotection in primary dopaminergic neuronal culture

**Figure 1:**
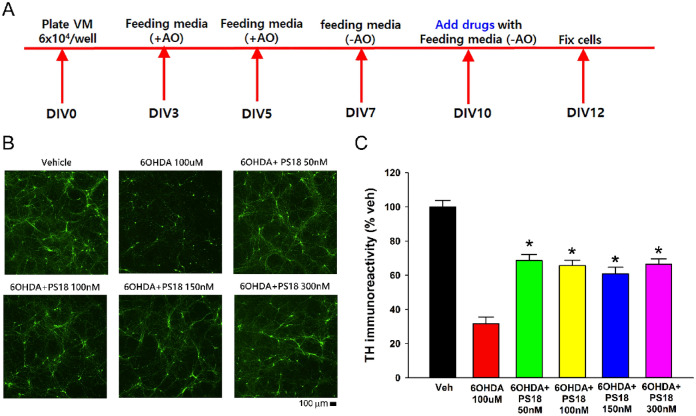
PS18 antagonized 6-OHDA -mediated dopaminergic neuronal loss in rat primary ventromesencephalic culture. (**A**) Timeline of experiment. (**B**) Representing photomicrographs demonstrate that 6-OHDA reduced tyrosine hydroxylase immunoreactivity (THir), which was antagonized by PS18. (**C**) PS18 significantly increased THir after 6-OHDA lesioning. n = 7 per group. *p < 0.05, 1-way ANOVA + NK test.

Ventral mesencephalon (VM) tissues were harvested from rat fetal brains for primary dopaminergic neuronal culture. 6-OHDA, PS18, and vehicle were added to the culture on DIV10 (day-in-vitro 10, Fig. 1A). As seen in the representing photomicrographs (Fig. 1B), 6-OHDA reduced the density of TH ( +) cell body and fibers. TH immunoreactivity (TH-ir) was averaged from all culture wells (n = 7 in each group). 6-OHDA significantly reduced TH-ir, which was significantly antagonized by PS18 (Fig. 1B and C).

### PS18 antagonized 6-OHDA -mediated TUNEL in rat primary dopaminergic culture

**Figure 2:**
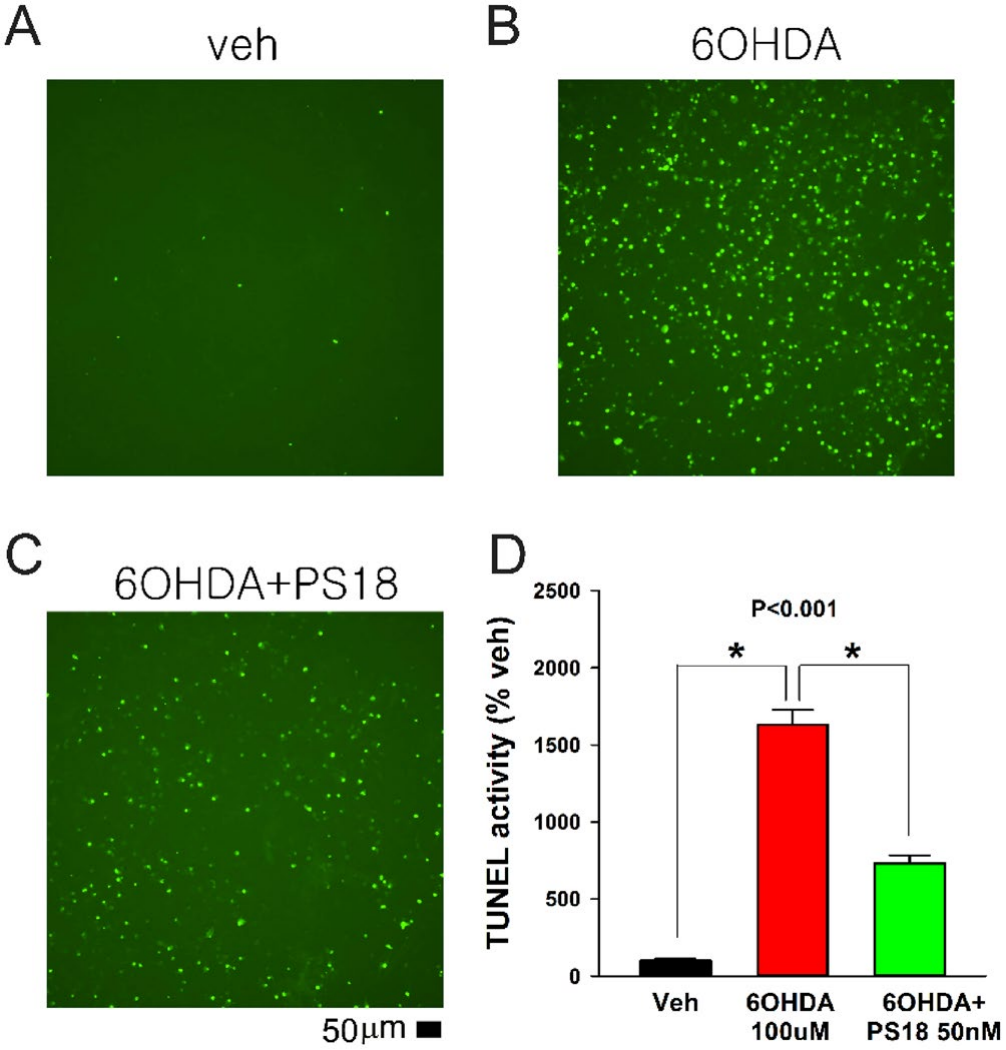
PS18 antagonized 6-OHDA -mediated TUNEL in rat primary dopaminergic culture. Representing photomicrographs demonstrate that 6-OHDA (100 μM) induced TUNEL. PS18 (50 nM) significantly reduced TUNEL ( +) cell density (*p < 0.001, 1-way ANOVA + NK test). Scale bar = 50 μm.

6-OHDA increased TUNEL (Fig. 2B vs. A) in primary dopaminergic culture; PS18 mitigated this reaction (Fig. 2C vs. B). In all culture studied, PS18 significantly antagonized 6-OHDA -activated TUNEL in VM cultures (Fig. 2D, *p* < 0.001, F_2,15_ = 140.177, 1-way ANOVA + NK test).

### PS18 antagonized thapsigargin (Tg) or methamphetamine -mediated apoptosis in primary dopaminergic neuronal culture

**Figure 3:**
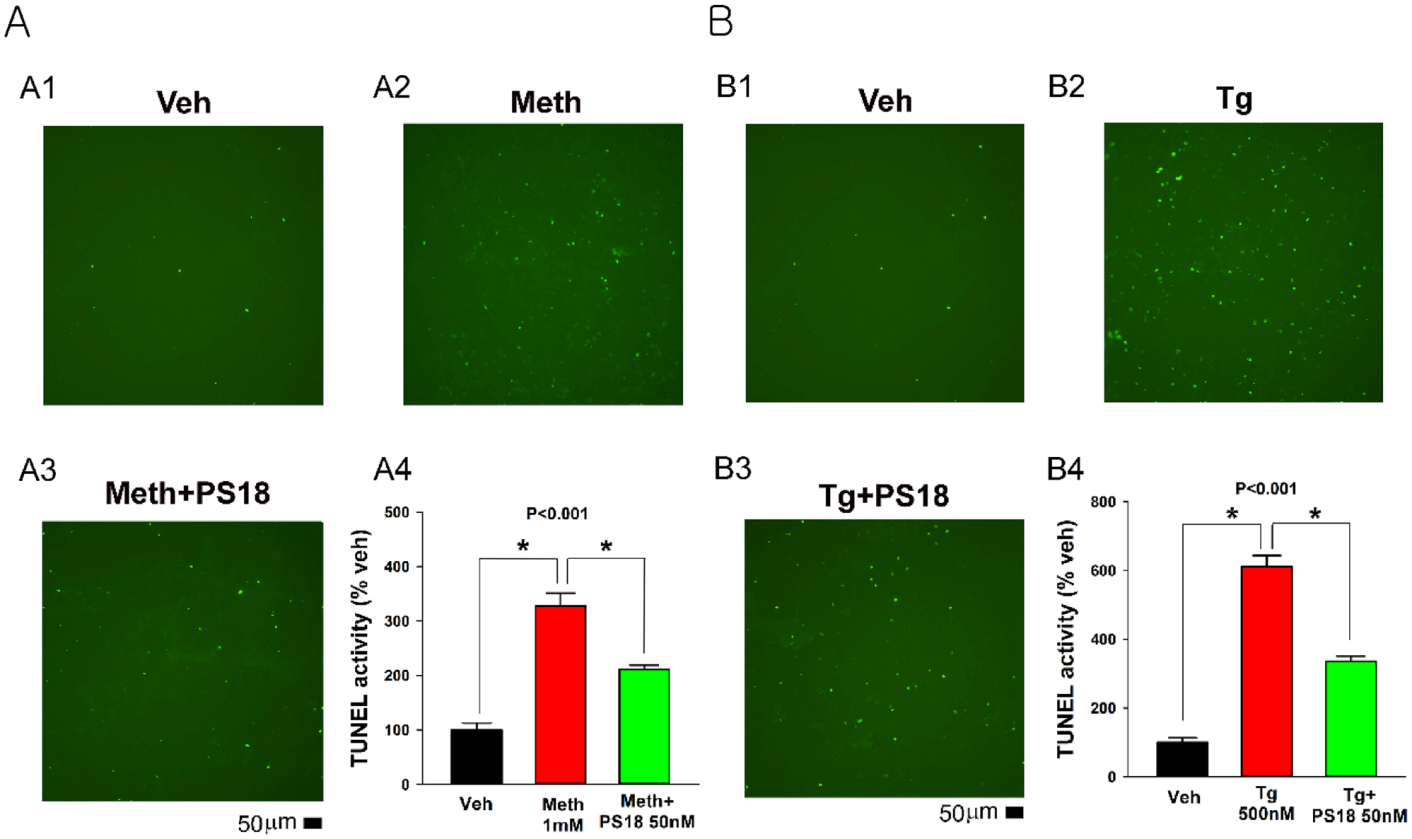
PS18 antagonized Meth and Tg-mediated apoptosis in rat primary dopaminergic culture. Representing photomicrographs demonstrate that Meth (1 mM) or Tg (500 nM) increased TUNEL in dopaminergic neuronal culture. PS18 (50 nM) significantly reduced TUNEL ( +) cell density (*p < 0.001, 1-way ANOVA + NK test). Scale bar = 50 μm.

ER stressors Tg or methamphetamine (Meth) were added to the VM culture. Both Tg and Meth increased TUNEL (Fig. 3). These responses were significantly antagonized by PS18 (Fig. 3A and B Meth: *p* < 0.001, F_2,15_ = 50.052; Tg: *p* < 0.001, F_2,15_ = 136.051, 1-way ANOVA + NK test).

### PS18 antagonized Tg or 6-OHDA-induced ER stress in SH‐SY5Y‐GLuc‐SERCaMP cell culture

**Figure 4:**
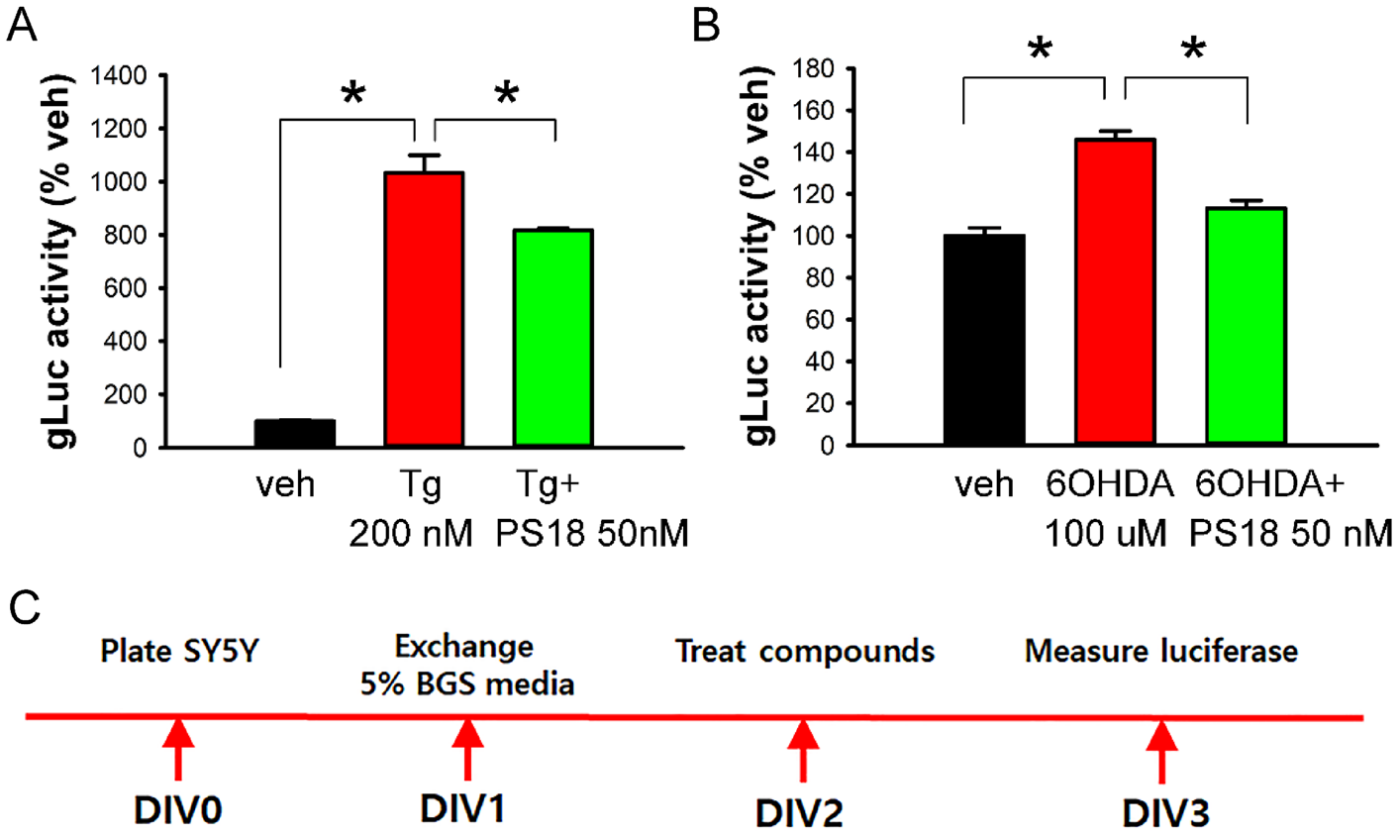
PS18 antagonized Tg or 6-OHDA-mediated ER stress in SY5Y cell cultures. gLuc activity was quantified after treatment with (**A**) Tg (200 nmol/L) or (**B**) 6-OHDA (100 μM) (**B**). Tg or 6-OHDA significantly increased gLuc activity. These responses were antagonized by PS18 (A. *p* < 0.001; B, *p* < 0.001, 1-way ANOVA + NK test). (**C**) Timeline.

Previous studies have demonstrated that Tg–mediated ER stress can be quantitatively measured through the change of GLuc activity in cultured SH-SY5Y cells overexpressing ER stress reporter GLuc-SERCaMP^12,17^. SH‐SY5Y‐GLuc‐SERCaMP cells (1.65 × 10^5^/well) were plated on DIV0. ER stress was induced by Tg (200 nM) and 6-OHDA (100 μM) on DIV 2. Media was collected before and 24 h after drug treatment for gLuc assay (Fig. 4C). Tg (n = 5), compared to veh (n = 5), significantly increased gLuc activity in media. Co-treatment PS18 (50 nM, n = 5) significantly antagonized Tg-mediated gLuc release (Fig. 4A; *p* < 0.001, F_2,12_ = 160.471). PS18 (50 nM, n = 6) also significantly antagonized 6-OHDA (n = 6) -induced gLuc activity in culture media (Fig. 4B, n = 6; *p* < 0.001, F_2,15_ = 37.246).

### Time-dependent expression of Prosaposin in the 6-OHDA-lesioned brain

**Figure 5:**
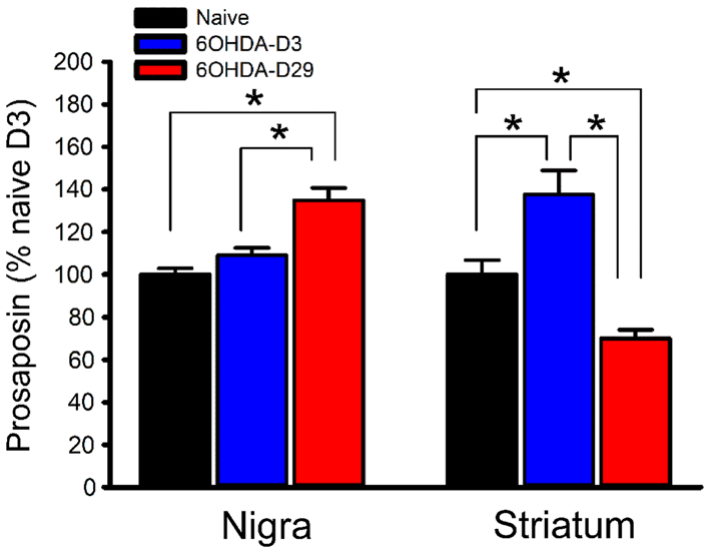
Upregulation of prosaposin in striatum and nigra after striatal 6-OHDA lesioning. Intra-striatal injection of 6-OHDA upregulated the expression of prosaposin in nigra (*p* < 0.001, F_2,16_ = 14.143, one-way ANOVA + NK test) and striatum (*p* < 0.001, 1-way ANOVA + NK test).

Fourteen rats received local 6-OHDA administration to the left striatum, as we described^18,19^. Another 5 rats were used as control. Animals were sacrificed 3 or 29 days after the lesioning or sham surgery. The expression of prosaposin in nigra and striatum was examined by qRTPCR. The expression of prosaposin was normalized to reference gene GAPDH (Table 1). Striatal lesioning transiently upregulated prosaposin in nigra (Fig. 5; naïve D3 vs. 6OHDA D29, *p* < 0.001; 6OHDA D3 vs. 6OHDA D29, *p* = 0.002, one-way ANOVA + NK test) and striatum (naïve D3 vs. 6OHDA D3, *p* = 0.005; naïve D3 vs. 6OHDA D29, *p* = 0.013; 6OHDA D3 vs. 6OHDA D29, *p* < 0.001, one-way ANOVA + NK test).

**Table 1:** Oligonucleotide primers used for quantitative RT-PCR.

Gene	SYBR green		TagMan
	Forward	Reverse	
Prosaposin	TAGGTCCATTTAACTGCACA	ACTTCTGAATGTAACTTGCC	
PERK	GAAGTGGCAAGAGGAGATGG	GAGTGGCCAGTCTGTGCTTT	
IRE1	TCATCTGGCCTCTTCTCTCGGA	TTGAGTGAGTGGTTGGAGGC	
Bip	TCGACTTGGGGACCACCTAT	GCCCTGATCGTTGGCTATGA	
ATF6	GGACCAGGTGGTGTCAGAG	GACAGCTCTGCGCTTTGGG	
TH			Rn00562500 _m1
β-Actin			Rn00667869_m1
GAPDH			Rn01775763_g1

### Prosaposin improved locomotor activity and reduced rotational behavior in unilaterally 6-OHDA-lesioned rats

**Figure 6:**
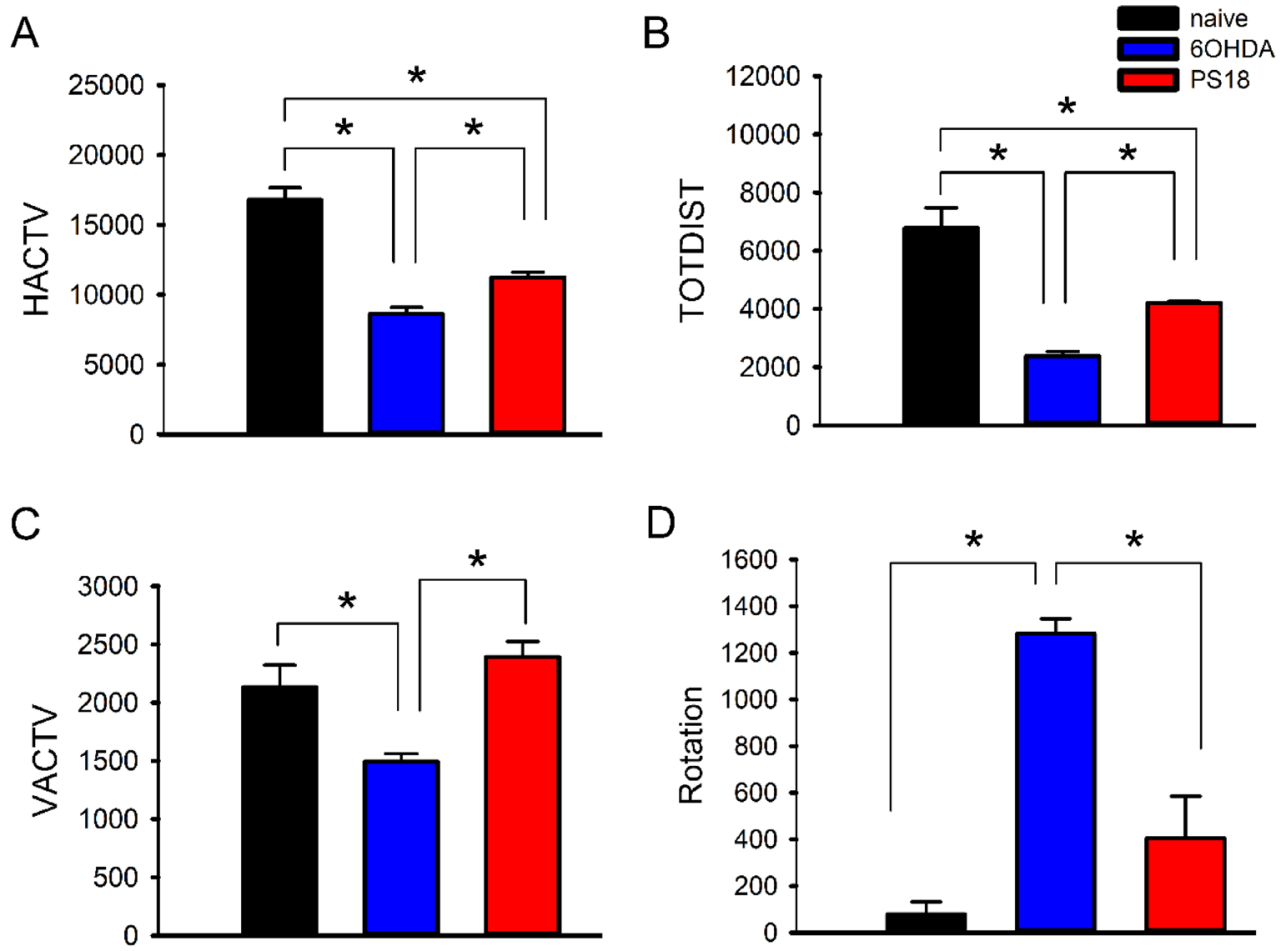
PS18 administration improved locomotor and reduced rotational behaviors in hemiparkinsonian rats. 6-OHDA was administered locally to the left striatum. Locomotor behavior was examined on D14. PS18 (i.c.v.) significantly increased (**A**) horizontal activity (HACTV), (**B**) total distance traveled (TOTDIST), and (**C**) vertical activity (VACTV). (**D**) Unilaterally 6-OHDA rats developed ipsilateral rotation on day 21 after challenging with low-dose methamphetamine; PS18 significantly mitigated rotation (*p < 0.05, 1-way ANOVA + NK test).

Adult rats (n = 8) received 6-OHDA infusion unilaterally. Of these animals, 4 received PS18 (2 mM/20 µL, i.c.v.) and 4 were treated with vehicle at 15 min prior to 6-OHDA lesioning. Control rats (n = 6) did not receive 6-OHDA or PS18. Locomotor activity was examined on day 14 (D14). 6-OHDA infusion significantly reduced horizontal activity (HACTV), total distance traveled (TOTDIST), and vertical activity (VACTV); these behavior responses were significantly antagonized by PS18 (Fig. 6, HACTV, *p* = 0.040; TOTDIST, *p* = 0.048; VACTV, *p* = 0.010, 1-way ANOVA + NK test).

Unilateral lesioning resulted in the imbalance of dopamine innervation and rotational behavior. As seen in Fig. 6D, the administration of indirect dopaminergic agonist Meth (2.5 mg/kg) induced ipsilateral rotation, up to 1,200 turns per 60 min on day 21. The rotation was significantly attenuated by PS18 (Fig. 6D, *p* < 0.001, F_2,9_ = 29.555, 1-way ANOVA + NK test).

### PS18 increased the expression of dopamine transporter DAT and prosaposin mRNA at the lesioned brain

**Figure 7:**
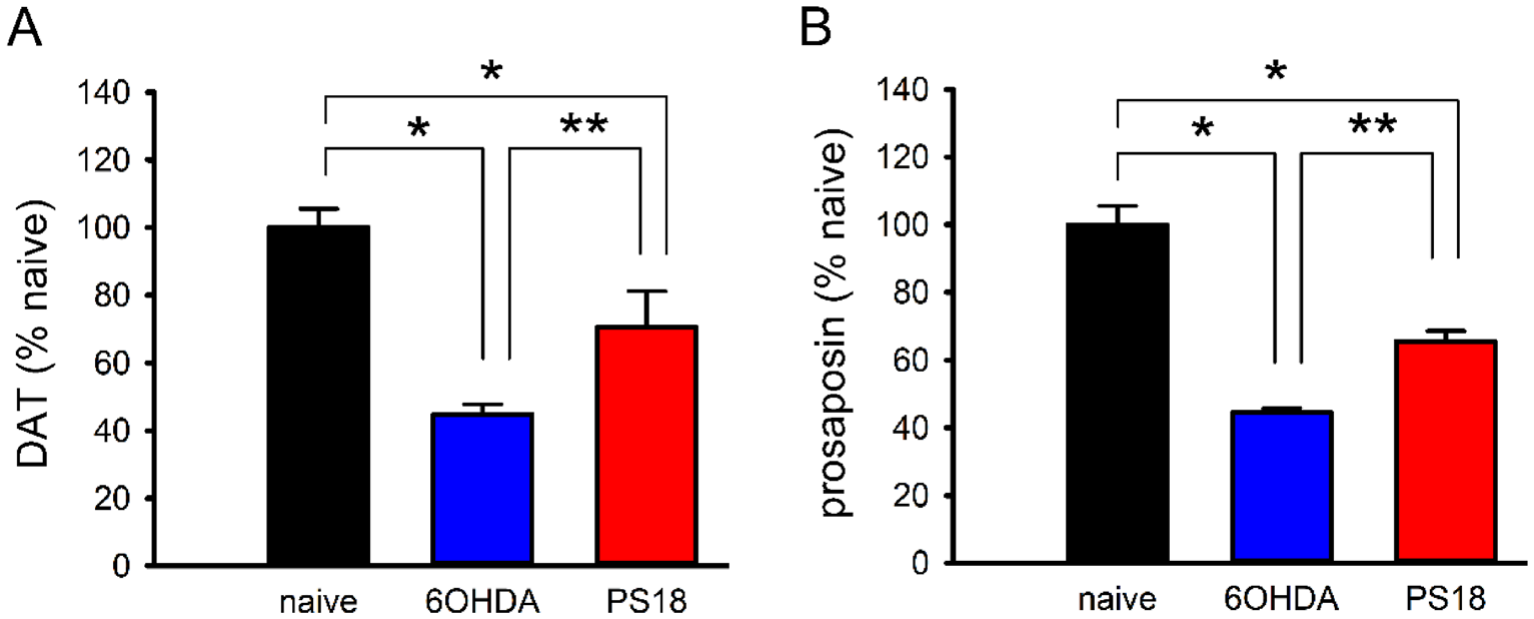
PS18 increased DAT and prosaposin expression in the lesioned striatum. Striatum tissues were collected on D29 after 6-OHDA lesioning. 6-OHDA significantly downregulated (**A**) DAT and (**B**) prosaposin; PS18 significantly antagonized these responses. *p < 0.05, 1-way ANOVA + NK test.

Striatal tissues were collected for qRTPCR analysis on D29 after 6-OHDA lesioning (naïve, n = 8; 6-OHDA + veh, n = 4; 6-OHDA + PS18, n = 4). PS18 or vehicle (saline) was administered intracerebroventricularly 15 min before 6-OHDA. 6-OHDA significantly downregulated DAT and prosaposin on D29. PS18 significantly increased the expression of DAT and prosaposin in the lesioned striatum (Fig. 7. DAT, *p* = 0.036; prosaposin, *p* = 0.028, 1-way ANOVA + NK test).

### PS18 suppressed the expression of ER stress markers in the 6-OHDA-lesioned nigra

**Figure 8:**
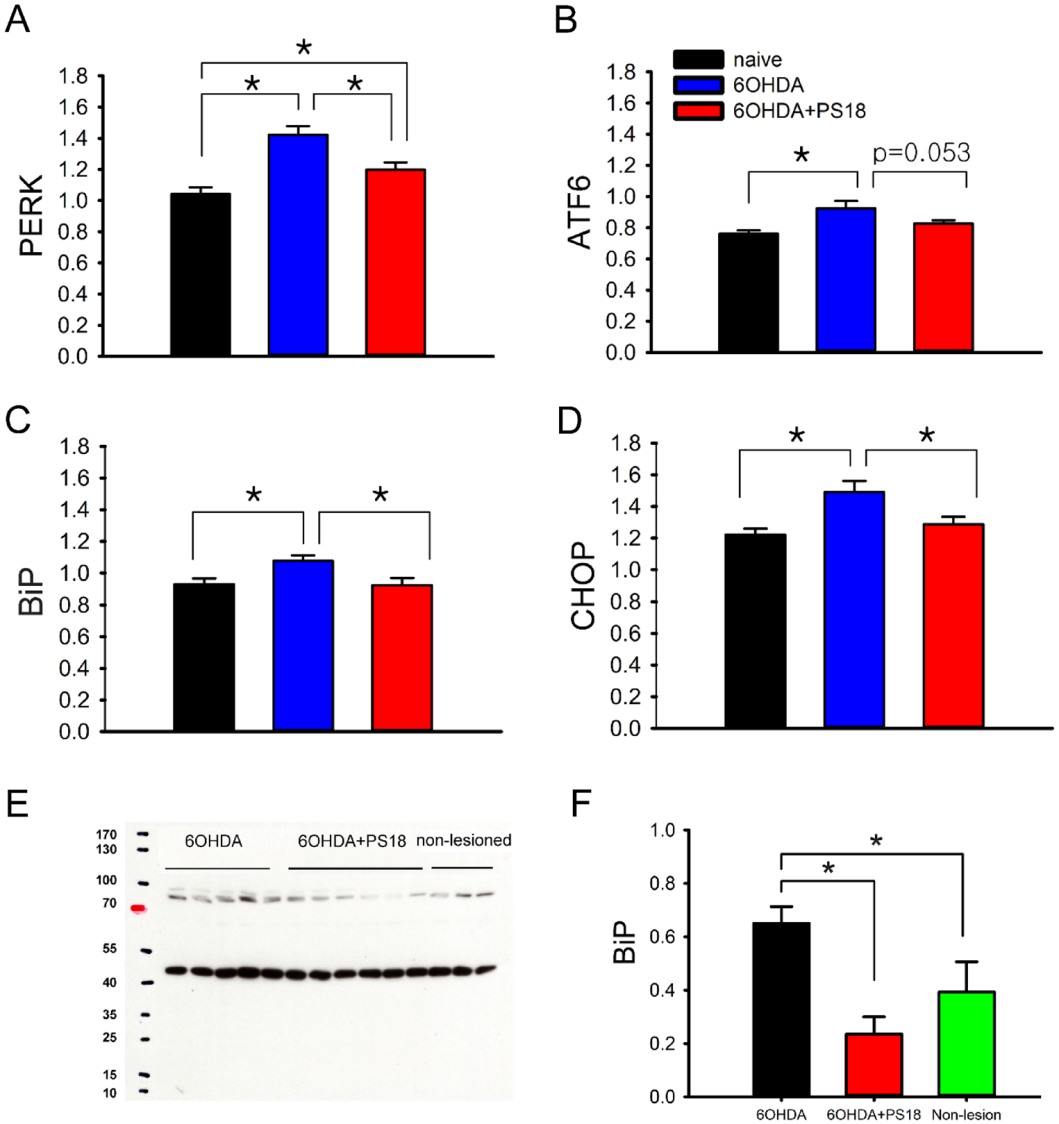
PS18 suppressed the expression of ER stress markers in nigra. (**A**–**D**) 6-OHDA significantly upregulated the expression of PERK, ATF6, CHOP, and BiP mRNA in the lesioned nigra, examined by qRTPCR. PS18 significantly attenuated the expression of (**A**) PERK, (**C**) BiP, and (**D**) CHOP. (E, F) Another set of animals were used for Western blot analysis. (**E**) Representing Western blots. (**B**) Administration of 6-OHDA significantly increased BiP protein level in the lesioned nigra (non-lesioned vs. 6-OHDA). PS18 antagonized 6-OHDA-induced BiP expression (*p* < 0.05, one-way ANOVA + NK test).

Nigra tissues from 17 rats (naïve, n = 7, 6-OHDA + veh, n = 5, 6-OHDA + PS18, n = 5) were collected 29 days after 6-OHDA lesioning. 6-OHDA significantly upregulated the expression of PERK, ATF6, CHOP, and BiP in the lesioned nigra, while PS18 antagonized these responses (Fig. 8, A: PERK, *p* = 0.008; C: BiP, *p* = 0.016, D: CHOP, *p* = 0.019, 1-way ANOVA). In addition, a trend toward significance was found in ATF6 (Fig. 8B, *p* = 0.053). In a parallel experiment, 14 rats were used for Western blot analysis (5 received 6-OHDA + vehicle, 6 received 6-OHDA + PS18, and 3 were used as non-lesioned control). Nigra tissues were collected on day 5 after 6-OHDA lesioning. BiP protein expression was significantly induced in the lesioned nigra. PS18 significantly antagonized 6-OHDA –induced BiP protein in nigra (Fig. 8E and F, *p* = 0.001, F_2,11_ = 9.213, 1-way ANOVA + NK test).

### PS18 increased TH immunoreactivity (TH-ir) in the lesioned nigra

**Figure 9:**
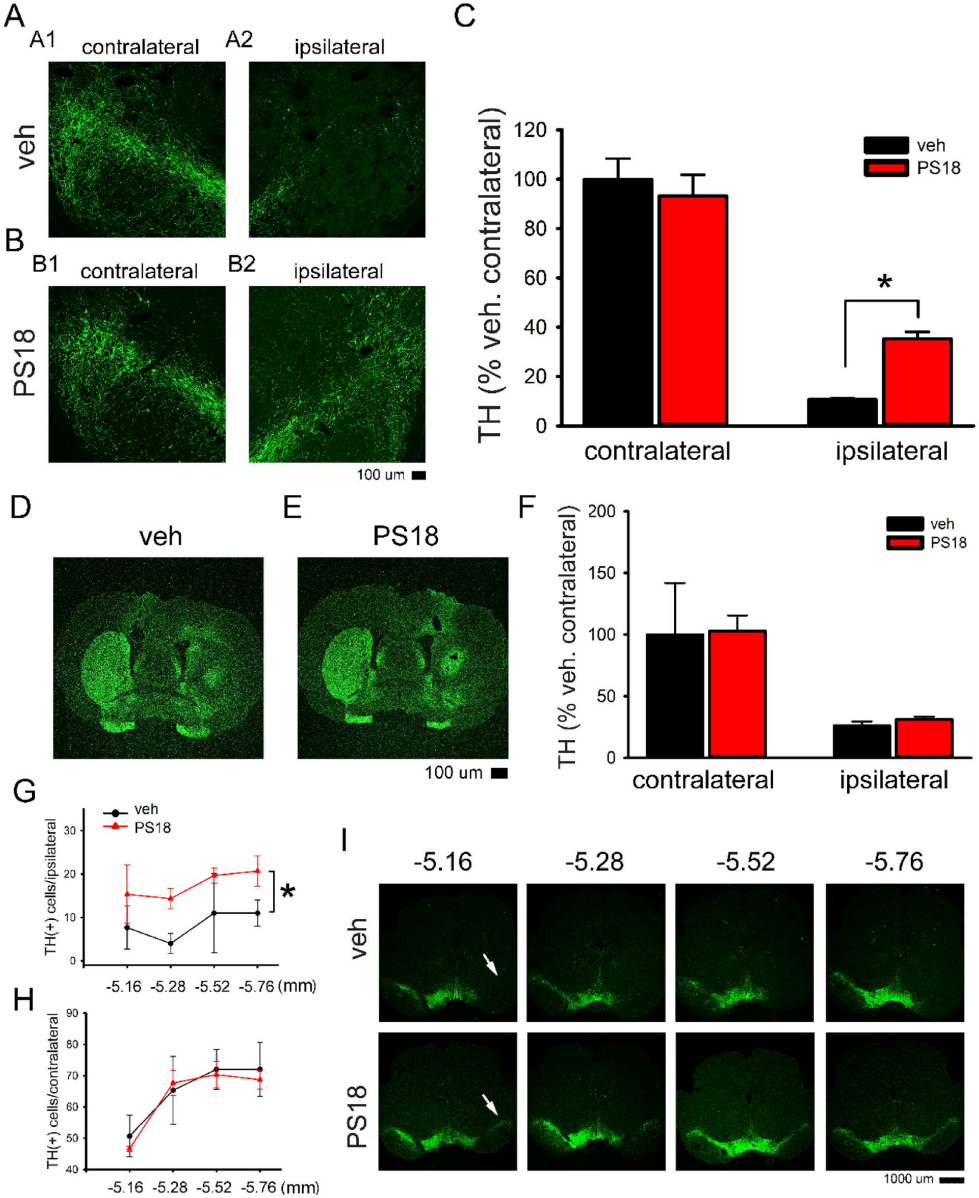
PS18 antagonized 6-OHDA–mediated dopaminergic neurodegeneration in nigra. (**A**) In a rat receiving vehicle, unilateral injection of 6-OHDA reduced TH-ir in the lesioned (ipsilateral) nigra (A2 vs. A1). (**B**) Intracerebroventricular administration of PS18 increased TH-ir in the lesioned nigra (B2 vs. A2). (**C**) TH-ir was quantified in all animals studied. PS18 significantly mitigated TH-ir loss in the lesioned nigra (*p* = 0.021, 1-way ANOVA + NK test). (**D**–**F**) PS18 did not alter dopaminergic neurodegeneration in the striatum. Typical TH immunostaining from animals receiving (**D**) vehicle or (**E**) PS18. (**F**) PS18 did not significantly alter TH activity in the lesioned striatum. (**G**) TH cells from 4 brain slices between bregma −5.16 and −5.76 mm per each animal. Only TH neurons with the presence of nuclei were counted. 6-OHDA significantly reduced TH( +) cells in the lesioned nigra (G vs. H, non-lesioned vs. 6-OHDA; *p* < 0.001, 2-way ANOVA + NK test). (**G**) PS18 significantly increased the TH cell number in the lesioned nigra (6-OHDA vs. 6-OHDA + PS18, *p* = 0.029, 2-way ANOVA + NK test). (**H**) PS18 did not alter TH cell number in the non-lesioned side nigra. (**I**) Representing TH immunoreactivity in the lesioned nigra (arrows) at lower magnification. Calibration = 100 μm (**A**–**D**) and 1000 μm (**H**).

Adult rats received vehicle (n = 3, saline) or PS18 (n = 3, 2 mM, i.c.v.) after unilateral 6-OHDA lesioning in striatum on D0. Animals were sacrificed and perfused 5 days after 6-OHDA lesioning. Brain tissues were collected for immunohistochemistry. Striatal TH-ir was quantified in all brain slices with visible anterior commissure in each rat. Nigral TH-ir was also examined between −5.52 and −5.64 mm. Lesioning with 6-OHDA significantly diminished TH-ir in the lesioned nigra (Fig. 9A1 vs. A2; *p* < 0.001, 1-way ANOVA + NK test) and almost abolished TH-ir in the ipsilateral striatum (Fig. 9D and E). PS18 did not significantly alter TH-ir in the lesioned striatum (Fig. 9F, *p* = 0.292), however significantly antagonized the loss of TH-ir in lesioned nigra (Fig. 9B2 vs. B1, and C, *p* = 0.021, F_3,8_ = 52.217, 1-way ANOVA + NK test). TH( +) cell numbers in nigra were calculated from 4 brain slices between Bregma −5.16 to −5.76 mm per each animal. Administration with 6-OHDA significantly reduced TH ( +) cells number in the lesioned nigra (Fig. 9G: lesioned vs. Fig. 9H non-lesioned, *p* < 0.001, 2-way ANOVA + NK test). PS18 significantly increased the TH cell number in the lesioned nigra (Fig. 9G and I; *p* = 0.029, 2-way ANOVA + NK test).

### PS18 increased TH protein level in lesioned nigra

**Figure 10:**
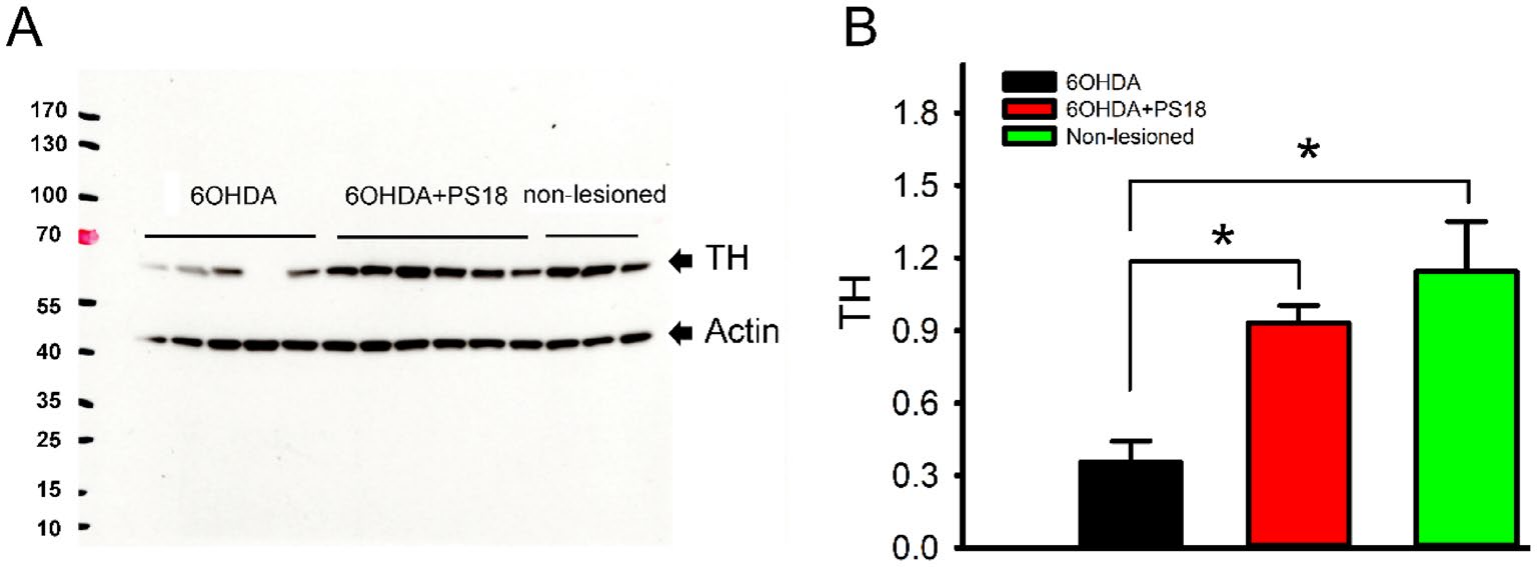
PS18 increased TH protein level in lesioned nigra. (**A**) Representing Western blots of TH protein in the lesioned (6-OHDA) nigra. (**B**) Administration of 6-OHDA significantly reduced TH protein level in the lesioned nigra (non-lesioned vs. 6-OHDA; *p* < 0.001, 1-way ANOVA + NK test). PS18 antagonized 6-OHDA-mediated TH loss in the lesioned nigra (6-OHDA vs. 6-OHDA + PS18, *p* = 0.001, 1-way ANOVA + NK test).

Fourteen rats were used for Western blot analysis (5 received 6-OHDA + vehicle, 6 received 6-OHDA + PS18, and 3 were used as non-lesioned control). Striatum and nigra were collected on day 5 after 6-OHDA lesioning. TH protein expression was significantly reduced in the lesioned striatum (*p* < 0.001) and nigra (*p* = 0.005, 1-way ANOVA + NK test). PS18 significantly antagonized 6-OHDA –mediated TH protein loss in nigra (Fig. 10A and B, *p* = 0.001, F_2,11_ = 14.011, 1-way ANOVA + NK test), but not in striatum (*p* = 0.220, 1-way ANOVA + NK test).

## Discussions

In this study, we reported that PS18 antagonized 6-OHDA -mediated dopaminergic degeneration in primary rat ventral mesencephalic neuronal culture. 6-OHDA significantly reduced TH immunoreactivity and increased TUNEL. These responses were antagonized by PS18. The protective effect of PS18 was further demonstrated in unilaterally 6-OHDA-lesioned rats. PS18 improved locomotor activity, reduced rotational behavior, upregulated striatal DAT expression, and increased nigral TH immunoreactivity in the lesioned animals. The major finding of our study is that PS18 is neuroprotective in cellular and animal PD models.

6-OHDA has been commonly used to examine the pathogenesis and therapy for PD^20^. 6-OHDA reduced mitochondrial activity, activated caspase-9 and caspase-3^21^, TUNEL, and programmed cell death, indicating that 6-OHDA induced neuronal degeneration through apoptosis. In this study, we showed that prosaposin PS18 reduced 6-OHDA-mediated TUNEL and the loss of TH-ir in dopaminergic neuronal culture. Our data thus support that PS18 induced protection by inhibiting apoptosis in dopaminergic neurons.

We demonstrated that striatal 6-OHDA lesioning time-dependently regulated the expression of prosaposin in nigra and striatum. 6-OHDA transiently increased the expression of striatal prosaposin on D3. A delayed upregulation of prosaposin on D29 was found in nigra. As prosaposin is protective against 6-OHDA, the upregulation of prosaposin may indicate time-dependent endogenous neuroprotection was turned on after lesioning striatum and nigra. However, this endogenous protection was insufficient as prosaposin mRNA was down-regulated in striatum on D29. Exogenous prosaposin PS18 was needed to antagonize the dopaminergic degeneration and improve the prosaposin expression on D29.

Midbrain dopaminergic neuron death is associated with motor impairment, including bradykinesia and difficulty initiating voluntary movement in PD^22–25^. Similar responses were found in the 6-OHDA -lesioned rats. Horizontal activity (HACTV), total distance traveled (TOTDIST), and vertical activity (VACTV) were significantly reduced in hemiparkinsonian rats. The unilaterally 6-OHDA -lesioned rats also developed ipsilateral rotation in response to dopaminergic agonists^26^. In this study, PS18 significantly antagonized bradykinesia and low-dose methamphetamine-induced ipsilateral rotation 14 and 21 days after 6-OHDA lesioning. Altogether, these data suggest that PS18 improved motor behavior in hemiparkinsonian rats.

Using immunohistochemistry, we demonstrated that 6-OHDA significantly reduced TH immunoreactivity and cell number in nigra. Both responses were significantly antagonized by PS18. The protective effect of PS18 in nigra was further supported by the Western blot analysis. We demonstrated that TH protein expression was significantly reduced in the lesioned nigra, and PS18 significantly antagonized 6-OHDA –mediated TH protein loss in nigra. Our data suggest that PS18 partially preserved TH activity in the nigra and PS18 is neuroprotective against dopaminergic neuronal loss in nigra. We also found that PS18 improved the expression of DAT mRNA in lesioned striatum on day 29. However, it did not significantly alter striatal TH protein expression on day 5. The discrepancy of dopaminergic marker expression in striatum may be attributed to the difference in protein translation or the time-dependent protection in striatum, which warrant future study.

ER stress is a major cause of dopaminergic degeneration in PD^27^. 6-OHDA-induced degeneration through ER stress^28^. We demonstrated that 6-OHDA significantly upregulated the expression of ER stress markers (i.e., PERK, ATF6, CHOP, and BiP) in the lesioned nigra. 6-OHDA also significantly increased BiP protein production in nigra. These responses were antagonized by PS18. To further characterize the interaction of PS18 and ER stress, SY5Y cells expressing ER stress reporter gLuc- SERCaMP were used^11,12^. PS18 significantly reduced Tg and 6-OHDA -mediated gLuc-SERCaMP release. Furthermore, Tg or 6-OHDA-mediated TUNEL was significantly antagonized by PS18 in dopaminergic neuronal culture. Altogether, these data suggest that the protective effect of PS18 against 6-OHDA-mediated neurodegeneration involves anti-ER stress.

We, and others, previously reported that mesencephalic astrocyte-derived neurotrophic factor (MANF) is responsive to ER stress^29^ and has been considered an ER stress response protein^30,31^. MANF protected against ER stress-induced cell death^30^. In addition, MANF reduced ER stress by facilitating the formation of cysteine bridges and protein folding in the ER^32^. As the N-terminal domain of MANF is structurally similar to a saposin, a common anti-ER stress pathway may be involved in MANF and prosaposin-mediated protection.

There are a few limitations in this study. TH cells were counted from 4 brain slices between bregma −5.16 to −5.76 mm per each animal. A more intensive unbiased stereology analysis is needed. Systemically applied prosaposin PS18 cannot cross the blood–brain barrier. In addition, large peptides are often quickly metabolized by protease in the periphery. To overcome these limitations, we applied PS18 intracerebroventricularly to 6-OHDA -lesioned rats. As i.c.v. is invasive and may not be clinically relevant, the effectiveness of PS18 through other delivery routes, such as intranasal or dioleoylphosphatidylserine liposome delivery system^5^, warrants further investigation.

In conclusion, we demonstrated that prosaposin PS18 induced neuroprotection against dopaminergic neurotoxin 6-OHDA in primary dopaminergic neuronal culture. Exogenous PS18 improved locomotor function, reduced rotational behavior, and improved nigral TH expression in hemiparkinsonian rats. The mechanisms of protection involve anti-apoptosis and anti-ER stress. Our data support the beneficial effects of prosaposin PS18nin PD animals. Further clinical studies are needed before clinical use.

## Materials and Methods

### Materials

Prosaposin PS18 was purchased from Elabscience (Texas, USA). Bovine serum albumin, fetal bovine serum, L-glutamate, NMDA, paraformaldehyde, polyethyleneimine, and Triton X-100, were purchased from the Sigma (St. Louis, USA). Alexa Fluor 488 (secondary antibody), B27 supplement, Dulbecco’s modified Eagle’s medium, Neurobasal Medium, and trypsin were purchased from Invitrogen (Carlsbad, USA). TH antibody was purchased from Millipore (Burlington, USA). In Situ Cell Death Detection Kit was purchased from Roche (Indianapolis, USA).

Adult male and time-pregnant Sprague–Dawley rats were purchased from BioLASCO, Taiwan. The use of animals was approved by the Animal Research Committee of the National Health Research Institutes of Taiwan (NHRI-IACUC-109097-M1). All animal experiments were carried out in accordance with the National Institutes of Health Guide for the Care and Use of Laboratory Animals (NIH Publications No. 8023, revised 1978).

### Primary cultures of rat ventral mesencephalon cells

Primary cultures were prepared from embryonic (E15) ventral mesencephalon (VM) tissues obtained from fetuses of timed-pregnant Sprague–Dawley rats. The whole brain was removed aseptically, and a small piece of tissue comprising the VM was dissected. After removing the blood vessels and meninges, pooled VM tissues were trypsinized (0.25%; Invitrogen, Carlsbad, CA) with gentle mixing for 15 min at 37 °C. After rinsing off trypsin with pre-warmed DMEM/F-12 (Invitrogen), cells were dissociated by trituration, counted, and plated into 96-well (6.0 × 10^4^/well) cell culture plates pre-coated with poly-D-lysine (Sigma-Aldrich). The culture plating medium consisted of Dulbecco’s modified Eagle medium/F12 supplemented with 10% heat-inactivated fetal bovine serum, 1 mM L-glutamine, and 2% B27 (Invitrogen). Cultures were maintained at 37 °C in a humidified atmosphere of 5% CO2 and 95% air. The cultures were fed by exchanging 50% of media with feed media (Neurobasal medium, Invitrogen) with 0.5 mM l-glutamate and 2% B27 with antioxidants supplement on DIV (days in vitro) 3 and 5.

### SH-SY5Y-GLuc-SERCaMP Cell culture

SH-SY5Y-GLuc-SERCaMP cells were kindly provided by Dr. Brandon Harvey of the National Institute on Drug Abuse i.r.p., NIH. Cells were cultured in a 37 °C humidified incubator with 5% CO_2_ in DMEM (4.5 g/L D-GLucose) containing 2 mM GlutaMAX, 10% bovine growth serum (Sigma Aldrich), 10 U/mL penicillin (Thermo Fisher Scientific), and 10 μg/mL streptomycin (Thermo Fisher Scientific). Cells were plated at 5 × 10^4^ cells per well (100 μL volume). Media were exchanged into DMEM (4.5 g/L D-GLucose) containing 2 mM GlutaMAX, 1.5% BGS, 10 U/mL penicillin and 10 μg/mL streptomycin before 16-h drug pre-treatment. Cells were incubated for 48 h prior to adding drugs. Media was collected (5 μL) prior to and at indicated time points post-drug treatment for enzymatic assay.

### Immunocytochemistry and quantitation

After removing the PFA solution, cells were washed with PBS, and the fixed cultures were treated for 1 h with blocking solution (2% BSA, 0. 1% Triton X-100 and 5% goat serum in PBS). The cells were incubated for 1 day at 4 °C with specific mono/polyclonal antibodies (i.e., TH) and then rinsed three times with PBS. The bound primary antibody was visualized using Alexa Fluor 488 secondary (Invitrogen). Images were acquired using a monochrome camera Qi1-mc attached to Nikon TE2000-E inverted microscope.

### Gaussia luciferase secretion assay

Five microliters of culture medium were transferred to white 96-well plates. Coelenterazine (Cat# 1-361204-200Regis Technologies) stock solutions were prepared at 20 mM in acidified methanol (10ul of 10 N HCl/1 ml of methanol) and stored at -80 °C as single-use aliquots. The prepared substrate was incubated at room temperature 30 min prior to use. One hundred microliters of the diluted substrate were injected into each well followed by immediate luminescence reading. The amount of luciferase was determined using a plate reader with an injector setup (Biotek Synergy HT, Winooski, VT) to immediately read the sample after injection. For secretion assays, vehicle controls were used in all experiments under conditions equivalent to the treatments.

### Terminal deoxynucleotidyl transferase (TdT)-mediated dNTP nick-end labeling (TUNEL)

Cultures were examined for DNA fragmentation using a TUNEL-based method (In Situ Cell Death Detection Kit; Roche, Indianapolis, IN). Briefly, 4% PFA fixed cells were permeabilized in 0.1% Triton X-100 in 0.1% sodium citrate for 2 min on ice. To label damaged nuclei, 50 μL of the TUNEL reaction mixture was added to each sample and kept at 37 °C in a humidified chamber for 60 min. Procedures for positive and negative controls were carried out as described in the manufacturer’s manual (Roche). Controls consisted of not adding the label solution (terminal deoxynucleotidyl transferase) to the TUNEL reaction mixture. Nikon TE2000 inverted microscope equipped with fluorescence was used to examine apoptosis.

### Drug administration

Animals were anesthetized with 3% isoflurane. PS18 (2 mM/20 μL) or vehicle (saline, 20 μl) was administered intracerebroventricularly (AP, − 0.8 mm; LV, − 1.5 mm; DV, − 3.5 mm) at 15 min before 6-OHDA lesioning through a Hamilton microsyringe. The injection speed was controlled by a syringe pump (Micro 4, WPI, Sarasota, FL).

### 6-OHDA lesioning

Animals were anesthetized with 3% isoflurane. 6-OHDA (3 μg/μl × 2.5 μl dissolved in 0.1% ascorbic acid) was stereotactically injected into the 2 sites of left striatum (AP, 1 mm; LV, 3.2 mm; DV, -6.1 mm below the skull and AP, 1 mm; LV, 2.6 mm; DV, − 5.5 mm below the skull) at 0.25 μl/min over a 10 min period.

### Behavioral test

1. Locomotor activity was examined on day 14 after 6OHDA lesioning. Rats were individually placed in 42 × 42 × 31 cm Plexiglas activity chambers containing horizontal and vertical infrared sensors (Accuscan, Columbus, OH) placed 2.5 cm apart. Three variables were measured: (i) horizontal activity (HACTV, the total number of beam interruptions that occurred in the horizontal sensors in one hour), (ii) vertical activity (VACTV, the total number of beam interruptions that occurred in the vertical sensor in one hour), and (iii) total distance traveled (TOTDIST, the distance, in centimeters, traveled by the animals in one hour).
2. Rotational behavior was evaluated using an 8-channel rotometer system (RotoMax, AccuScan Instruments, Inc). Meth (2.5 mg/kg)-induced rotation per hour was counted by a computer, as we previously described^33^.

### Quantitative reverse transcription-PCR

Nigra tissues from the lesioned and non-lesioned side hemispheres were collected. Total RNAs were isolated using TRIzol Reagent (ThermoFisher, #15,596–018, Waltham, MA, USA), and cDNAs were synthesized from 1 μg total RNA by use of RevertAid H Minus First Strand cDNA Synthesis Kit (Thermo Scientific, #K1631, Waltham, MA, USA). cDNA levels for PERK, ATF6, BIP, IRE1, actin, and GAPDH were determined by specific universal probe library primer–probe sets or gene-specific primers (Table 1). Samples were mixed with TaqMan Fast Advanced Master Mix (Life Technologies, #4,444,557, Carlsbad, CA, USA) or SYBR (Luminaris Color HiGreen Low ROX qPCR Master Mix; ThermoScientific, Waltham, MA, USA). Quantitative real-time PCR (qRT-PCR) was carried out using the QuantStudio™ 3 Real-Time PCR System (ThermoScientific, Waltham, MA, USA). The expression of the target genes was normalized relative to the endogenous reference gene (beta-actin and GAPDH averages) with a modified delta-delta-Ct algorithm. All experiments were carried out in duplicate.

### Immunohistochemistry

Animals were anesthetized and perfused transcardially with saline followed by 4% PFA in phosphate buffer (PB; 0.1 mol/L; pH 7.2); they were post-fixed for 18–20 h and then transferred to 20% sucrose in 0.1 M PB for at least 16 h. Serial sections of brains were cut at a 30-μm thickness on a cryostat (model: CM 3050 S; Leica, Heidelberg, Germany). Brain sections were rinsed in PB and blocked with 4% bovine serum albumin (Sigma-Aldrich) and 0.3% Triton X-100 (Sigma-Aldrich) in 0.1 mM PB. Brain slices were incubated with primary antibody against anti-TH (monoclonal 1:100, Abcam, Cambridge, UK) at 4 °C overnight. Sections were rinsed in 0.1 mM PB and incubated in Alexa Fluor 488 secondary antibody solution (1:500; Molecular Probes, Eugene, OR, USA). Control sections were incubated without the primary antibody. Brain sections were mounted on slides and coverslipped. Confocal analysis was performed using a Nikon D-ECLIPSE 80i microscope (Nikon Instruments, Inc., Tokyo, Japan) and EZ-C1 3.90 software (Nikon, Tokyo, Japan).

TH density (TH pixel density in striatum or nigra—the background density in cortex) was measured in brain sections with a visualized anterior commissure (for striatal TH) and in sections between −5.16 and −5.76 mm (for nigra). TH optical density was analyzed by NIS Elements AR 3.2 Software (Nikon) and was averaged in each brain for statistical analysis. TH( +) cell numbers in nigra were also examined in 4 brain sections between bregma −5.16 mm to −5.76 mm using NIS Elements AR 3.2 Software (Nikon). Only TH neurons with presence of nuclei were counted. All immunohistochemical measurements were performed by blinded observers.

### Western blotting

The brain tissues were homogenized in RIPA lysis buffer (Merck Millipore, MA, USA) and then centrifuged at 13,200 rpm for 10 min at 4 °C. The supernatant was collected. A bicinchoninic acid (BCA) protein assay was performed to determine protein concentrations. The samples were diluted according to the BCA protein assay. Gels were transferred to a PVDF membrane after electrophoresis. The membranes were blocked in 5% milk at room temp for 1 h. The blots were then probed with primary antibodies against tyrosine hydroxylase (polyclonal, TH, 1: 10,000, Millipore, MA, USA), BiP (polyclonal, 1:1000, Cell Signaling, MA, USA), and actin (monoclonal, 1: 10,000, Novus, CO, USA) at 4 °C for overnight. The membrane was then incubated with horseradish peroxidase (HRP)-conjugated secondary antibody (Jackson lab) at room temp for 1 h, followed by washing with 0.1% Tween-20 (in PBS) three times for 10 min each. The light emission signal of the target proteins on the PVDF membrane was generated using a Western Lightning Plus-ECL (PerkinElmer, MA, USA) and then detected by X-ray film (Cat. No. GE28-9068–39, GE, Boston, USA). The amount of TH was normalized with actin on the same membrane. Band intensity was quantified using Image J.

### Statistical analysis

Data are presented as the mean ± SEM. Unpaired t-test, one- or two-way ANOVA, and post-hoc Newman–Keuls (NK) test were used for statistical comparisons, with a significance level of *p* < 0.05.

### Ethical approval

All procedures and animal experiments were fully complied with the Animal Research Committee of the National Health Research Institutes of Taiwan (NHRI-IACUC-109097-M1). All animal experiments were carried out in accordance with the National Institutes of Health guide for the care and use of Laboratory Animals (NIH Publications No. 8023, revised 1978). This study is reported in accordance with ARRIVE guidelines.

## Data Availability

The datasets generated or analyzed in the current study are available from the corresponding authors on reasonable request (contact: b7508@nhri.edu.tw).

## Abbreviations

6-OHDA: 6-Hydroxydopamine
ATF6: Activates transcription factor 6
BiP: Binding-immunoglobulin protein aka GRP-78
CHOP: CCAAT enhancer-binding protein homologous protein
ER: Endoplasmic reticulum
HACTV: Horizontal activity
PERK: Protein kinase RNA-like endoplasmic reticulum kinase
PD: Parkinson’s disease
PS18: Prosaposin-derived 18-mer peptide
qRTPCR: Quantitative reverse transcription PCR
Tg: Thapsigargin
TOTDIST: Total distance traveled
TUNEL: Terminal deoxynucleotidyl transferase (TdT)-mediated dNTP nick end labeling
VACTV: Vertical activity
VM: Ventral mesencephalon
